# Risk-based individualisation of target haemoglobin in haemodialysis patients with renal anaemia in the post-TREAT era: theoretical attitudes versus actual practice patterns (MONITOR-CKD5 study)

**DOI:** 10.1007/s11255-015-0970-8

**Published:** 2015-04-17

**Authors:** Loreto Gesualdo, Christian Combe, Adrian Covic, Frank Dellanna, David Goldsmith, Gérard London, Johannes F. Mann, Philippe Zaoui, Matthew Turner, Mike Muenzberg, Karen MacDonald, Ivo Abraham

**Affiliations:** Università degli Studi di Bari, Bari, Italy; Centre Hospitalier de Bordeaux and Unité INSERM 1026, University of Bordeaux, Bordeaux, France; G.I. Popa University Hospital of Medicine and Pharmacy, Iasi, Romania; Dialysezentrum, Düsseldorf, Germany; Guy’s and St Thomas’ NHS Foundation Hospital, London, UK; Centre Hospitalier F.H. Manhés, Fleury-Mérogis, France; Friedrich Alexander Universität Erlangen-Nürnberg, Erlangen, Germany; Université de Grenoble, Grenoble, France; Sandoz International GmbH, Industriestr. 25, 83607 Holzkirchen, Germany; Matrix 45, Tucson, AZ USA; University of Arizona College of Pharmacy, Tucson, AZ USA

**Keywords:** Anaemia, Biosimilar epoetin alfa, Congruence, Guidelines, Haemodialysis, Hb targets

## Abstract

**Purpose:**

Data from an ongoing European pharmacoepidemiological study (MONITOR-CKD5) were used to examine congruence between physician-reported risk-based individualisation of target haemoglobin (Hb) and the actual Hb targets set by these physicians for their patients, as well as actual Hb levels in their patients.

**Methods:**

Physician investigators participating in the study completed a questionnaire about their anaemia practice patterns and attitudes post-TREAT at the start of the study (T1) and in summer 2013 (T2). These data were compared with the Hb targets identified at baseline for actual patients (*n* = 1197) enrolled in the study. Risk groups included presence/absence of hypertension, diabetes, cardiovascular complications, history of stroke, history of cancer, and age/activity level (elderly/inactive or young/active).

**Results:**

At each time point, more than three quarters of physicians responded that results from the TREAT study, in patients not on dialysis, have influenced their use of erythropoiesis-stimulating agents in patients on haemodialysis. At T1, there was a clear difference in physician-reported (theoretical) target Hb levels for patients across the different risk groups, but there was no difference in patients’ actual Hb levels across the risk groups. A similar disparity was noted at T2.

**Conclusions:**

Physicians’ theoretical attitudes to anaemia management in patients on haemodialysis appear to have been influenced by the results of the TREAT study, which involved patients not on dialysis. Physicians claim to use risk-based target Hb levels to guide renal anaemia care. However, there is discrepancy between these declared risk-based target Hb levels and actual target Hb levels for patients with variable risk factors.

## Introduction

Anaemia is a common complication of chronic kidney disease (CKD), particularly among patients with stage 5 CKD who require renal replacement therapy [1]. Evidence-based guidelines recommend that the target haemoglobin (Hb) level to be achieved in patients with CKD by treatment with erythropoiesis-stimulating agents (ESAs) should be personalised based on an individual patient’s risk factors and risk–benefit analysis [2, 3].

The TREAT study, in patients with type 2 diabetes, CKD stage 3/4 (i.e. not receiving haemodialysis), and anaemia, randomised more than 4000 patients to one of two treatment arms: darbepoetin alfa to achieve a Hb level of approximately 13 g/dL or placebo, with rescue darbepoetin alfa when the Hb level was <9.0 g/dL [4]. The primary end points were the composite outcomes of death or a cardiovascular event and of death or end-stage renal disease. Treatment with darbepoetin alfa did not reduce the risk of either of the two primary composite outcomes [4]. However, pre-specified secondary analyses, although possibly underpowered [5], identified concerns related to the use of ESAs. Treatment with darbepoetin alfa was associated with a higher risk of cancer death among patients with a history of cancer at baseline, as well as an increased risk of fatal or non-fatal stroke (which appeared to be higher in patients with a history of stroke at baseline [4, 6]).

These findings understandably caused concern among the medical community and caused regulatory bodies in the USA (Food and Drug Administration) and Europe (European Medicines Agency) to recommend a more cautious approach to ESA use in the whole CKD population. Conversely, some scientists have questioned the validity of extrapolating data from TREAT and other studies in CKD stage 3/4 patients to the haemodialysis population [5, 7].

In light of the reaction to TREAT, as well as the more recent changes in the European labels for ESAs that set target Hb levels in the 10–12 g/dL range, we used data from the ongoing MONITOR-CKD5 study [8] to examine the congruence between physician-reported risk-based individualisation of target Hb and the actual Hb targets set by these physicians for their patients, as well as actual Hb levels obtained in their patients. We also report data from this study on Hb outcomes and dosing stability with biosimilar epoetin alfa (Binocrit^®^, Sandoz).

## Methods

MONITOR-CKD5 is a European pharmacoepidemiological study examining the multi-level determinants, predictors, and clinical outcomes of biosimilar epoetin alfa (Binocrit^®^) in haemodialysis patients. Full details of the study design have been reported previously [8]. Included patients are male or female adults on chronic haemodialysis due to end-stage renal disease (CKD stage 5), diagnosed with renal anaemia, and converted to anaemia management with biosimilar epoetin alfa.

For the present analysis, physician investigators participating in the MONITOR-CKD5 study were queried about their anaemia practice patterns and attitudes post-TREAT. The questionnaire was completed at the start of the study as centres were opened and patient enrolment began (T1, starting in 2009), and (per later decision) repeated with some modifications in summer 2013 (T2). The second time point was chosen to allow for the diffusion of the TREAT results and to capture the impact of its secondary findings. In particular, information was gathered on their target Hb values for various risk groups of patients receiving haemodialysis (hypothetical targets). These data were compared with the Hb targets identified at baseline for actual patients (*n* = 1197) enrolled in the study (patient targets). Hb targets were categorised as <10, 10–10.9, 11–11.9 and ≥12 g/dL. Risk groups included presence/absence of hypertension (HTN), diabetes, cardiovascular complications, history of stroke, history of cancer, and age/activity level (elderly/inactive or young/active). For Hb outcomes and dosing stability, data were evaluated based on the first 18 monthly visits (*n* = 2087 at baseline).

## Results

### Anaemia management and practice patterns

**Fig. 1:**
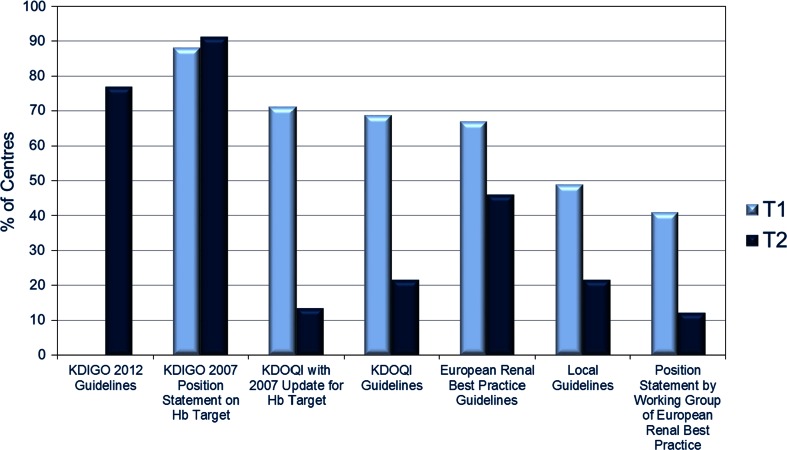
Centre adoption of clinical practice guidelines for anaemia management in patients with CKD (categories not mutually exclusive). *KDIGO* Kidney Disease-Improving Global Outcomes, *KDOQI* Kidney Disease Outcomes Quality Initiative

Centre and physician characteristics are shown in Table 1. Although fewer centres/physicians participated at T2, the characteristics were similar to those who participated at T1. The different guidelines followed by those physicians questioned are shown in Fig. 1. There was a drop in the proportion of centres adopting the Kidney Disease Outcomes Quality Initiative and European Renal Best Practice guidelines between T1 and T2. At T2, ~80 % of centres had adopted the updated Kidney Disease-Improving Global Outcomes (KDIGO) guidelines (these had not been published at T1). There was a statistically significant shift in the anaemia practice guidelines adopted by centres from T1 to T2 (*p* < 0.001). At T2, the KDIGO 2007 position statement and 2012 guidelines prevailed, and there was a continued modest adoption of the European Renal Best Practice guidelines. At both T1 and T2, most centres relied on more than one guideline.

**Table 1:** Physician and centre characteristics

	T1	T2
Centre		
N	109	74
Academic (%)	13.3	15.9
Academic affiliated (%)	11.4	12.1
Non-academic (%)	75.3	72.0
Mean (±SD) number of patients dialysed per week	101.0 (±59.9)	106.8 (±64.8)
Physician		
N	166	97
Male/female (%)	69.7/30.3	66.3/33.7
Mean (±SD) age (years)	49.4 (±9.0)	49.7 (±8.2)
Mean (±SD) years of practice	23.6 (±9.1)	24.2 (±8.8)

At each time point, more than three quarters of physicians responded that results from the TREAT study, in patients not on dialysis, have influenced their use of ESAs in patients on haemodialysis (T1, 77 %; T2, 78 %). There was no difference in the theoretical Hb threshold for ESA use (10 g/dL at both time points) and blood transfusions (7.4 g/dL at T1, 7.5 g/dL at T2). There was a lower ‘universal’ target Hb at T2 compared with T1 (40 % reported a target of <11.0 g/dL at T2, compared with 27 % at T1).

**Fig. 2:**
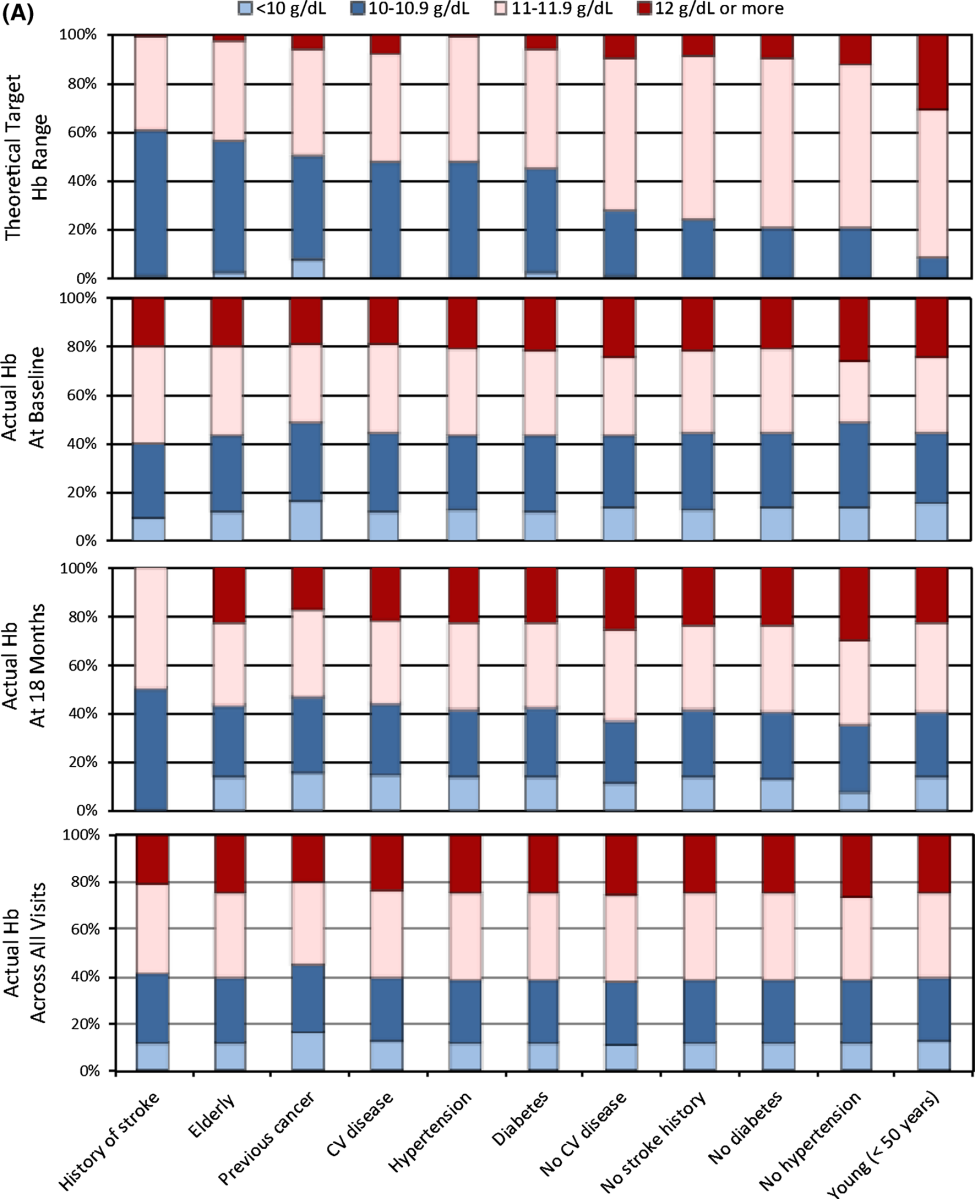

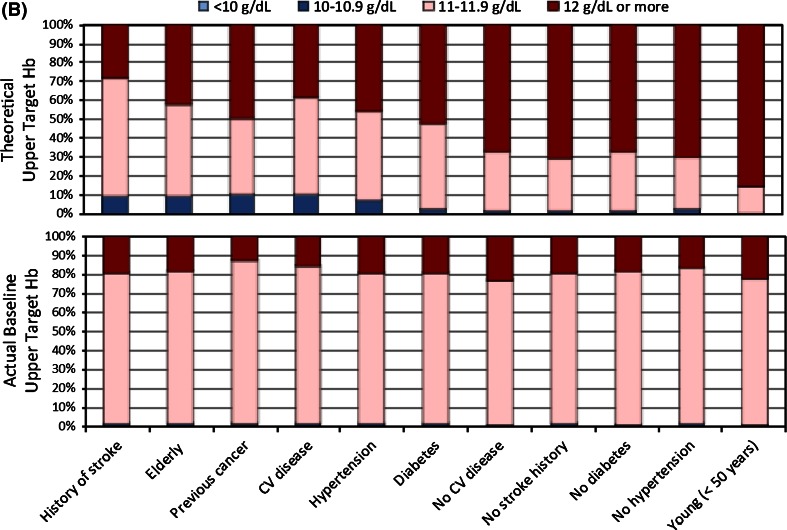
**a** Theoretical target Hb range at T1 and actual Hb levels at baseline, at 18 months, and across all visits. **b** Theoretical upper target Hb at T2 and actual upper target Hb at baseline

Figure 2 depicts for T1 (panel A) and T2 (panel B) the physician-reported (theoretical) target Hb and patients’ Hb levels, stratified by risk groups. Presented for T1 are the theoretical target Hb ranges and actual Hb levels at baseline, at 18 months, and across all visits (with risk factors listed in descending order of the proportion of physicians reporting a theoretical Hb target ≤10.9 g/dL for a given risk factor). As the upper graph in panel A shows, proportionately more physicians endorsed theoretical Hb targets ≤10.9 g/dL for the six risk groups of stroke, elderly, cancer, cardiovascular disease, HTN, and diabetes. In comparison, relatively more physicians reported using theoretical Hb targets ≥11 g/dL in patients without these risk factors. In contrast, patients’ actual Hb levels at baseline, at 18 months, and across all visits were similar across risk groups. Consistently across these three data points, at least 50 % of patients had Hb levels of 11 g/dL or more, and (with a few exceptions) at least 20 % of patients had Hb levels 12 g/dL or higher.

Figure 2b illustrates theoretical upper Hb targets at T2 versus patients’ baseline upper target Hb, by risk factor. Between 29.0 % (for the risk factor history of stroke) and 85.5 % (for the risk factor age <50 years) of physicians endorsed theoretical upper Hb targets of 12 g/dL or more. In contrast, the second graph in panel B shows that for about 80 % of patients a Hb level below 12 g/dL was specified as the target Hb to be pursued and, conversely, that only for about 20 % of patients a Hb level ≥12 g/dL was envisioned.

**Fig. 3:**
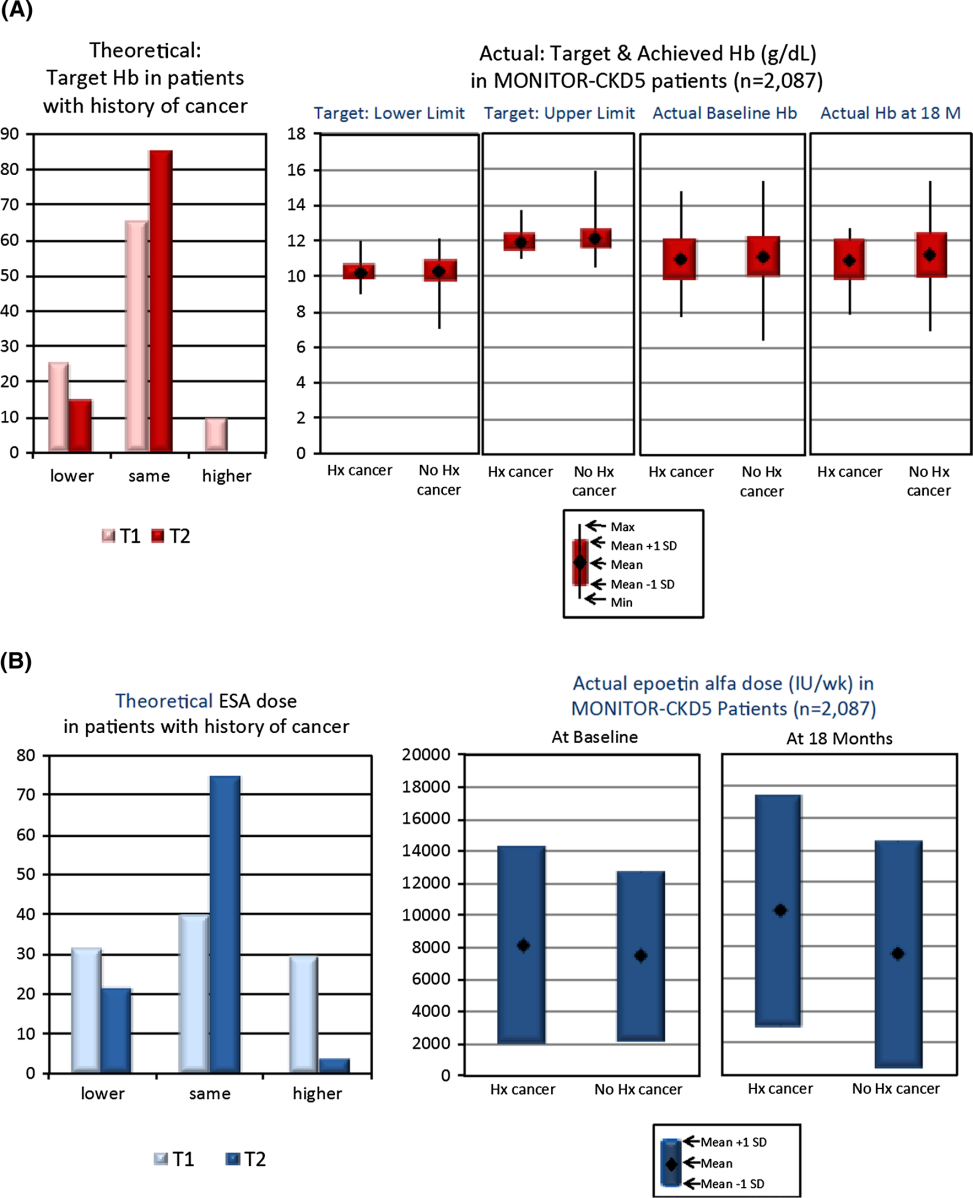
Anaemia management approach and practices in patients with and without a history of cancer. **a** Hb targets. **b** ESA dose. *ESA* erythropoiesis-stimulating agent, *Hb* haemoglobin, *Hx* history

When questioned specifically on their anaemia management approach in patients with a history of cancer (of specific interest following the TREAT study), 47 and 49 % of respondents at T1 claimed to consider time in remission and cancer burden when setting target Hb and ESA dose, respectively; 32 % answered that cancer history was not relevant. At T2, time in remission and cancer burden was considered relevant by 45 and 62 % of respondents, respectively; 25 % considered cancer history as not relevant. Physicians were also questioned about theoretical Hb targets and ESA doses in patients with a history of cancer, compared with those with no history of cancer (Fig. 3). For Hb, at T1, 25 % had a lower theoretical target, 65 % had the same target, and 10 % had a higher Hb target; at T2, 15 % had a lower Hb target and 85 % had the same target. For ESA dose, at T1, 31 % had a lower theoretical dose, 40 % had the same dose, and 29 % had a higher dose; at T2, 22 % had a lower theoretical dose, 74 % had the same dose, and only 4 % had a higher ESA dose. When actual Hb targets and ESA doses used were assessed for those with and without a history of cancer, no differences were found between the two groups (Fig. 3) (all *p* = ns).

**Fig. 4:**
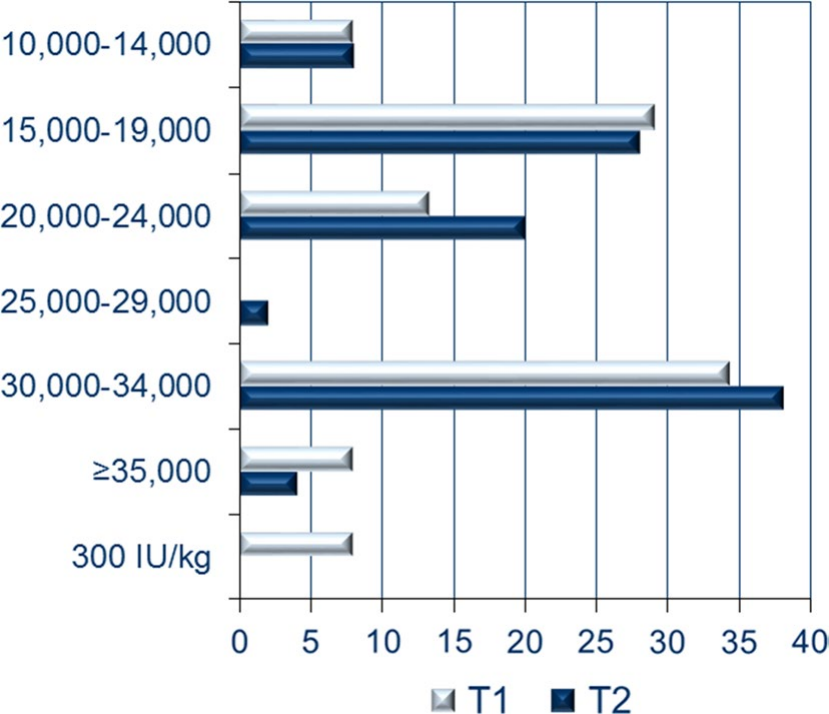
Distribution of maximum weekly ESA doses among physicians who responded that they do have an upper limit that should not be exceeded (doses are IU except where noted). *ESA* erythropoiesis-stimulating agent

At T1, 47 % of physicians reported that they did have an upper limit for weekly ESA dose that they would not exceed. At T2, this proportion increased to 74 %. The distribution of these maximum weekly doses is shown in Fig. 4. At T2, more than 40 % of respondents identified an upper limit of 30,000 IU/week or more.

**Fig. 5:**
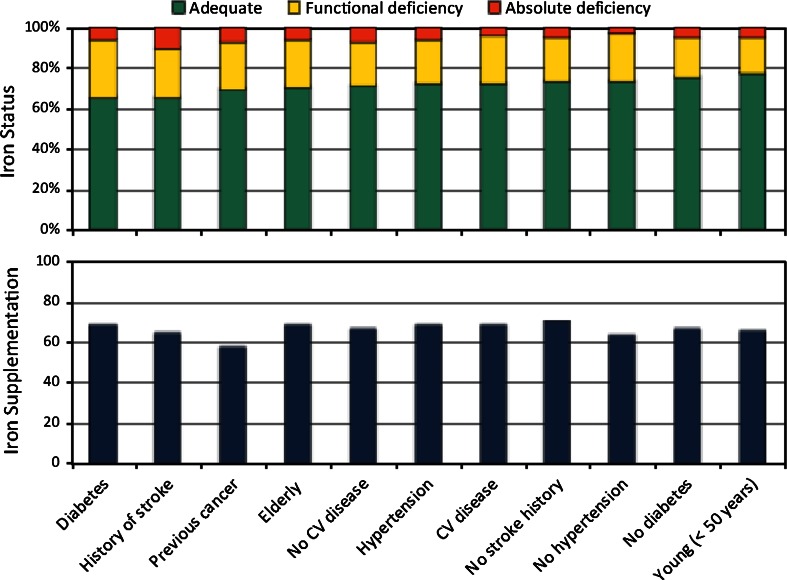
Iron status and iron supplementation by risk group

The survey also included questions on iron management practices. At T1, 81 % responded that they did have an upper target for serum ferritin that they would not intentionally exceed. Among these physicians, this upper target was 500 µg/L for 52 %, 750 µg/L for 30 %, and ≥1000 µg/L for 18 %. A shift towards a higher upper target for serum ferritin was noted at T2. At T2, 96 % of physicians replied that they had an upper serum ferritin target; among this group, the upper target was 500 µg/L for 29 %, 750 µg/L for 38 %, and ≥1000 µg/L for 33 %. Actual iron status by risk group is shown in Fig. 5. No significant differences in iron store by risk group pairs (e.g. between those with HTN and those without HTN) were found, except for diabetes; diabetics had a significantly lower rate of adequate iron stores than non-diabetics (65.5 vs. 75.8 %, *χ*^2^ = 8.24, *p* = 0.0162). Iron supplementation by risk group is also shown (Fig. 5); no significant differences in risk group pairs were found.

Iron status and supplementation were also explored by region (data not shown). Patients in Eastern Europe (EE) had significantly higher rates of adequate iron compared with patients in Western Europe (WE) (80.1 vs. 69.8 %, *χ*^2^ = 36.87, *p* < 0.0001), yet there was no significant difference in iron supplementation rates (62.7 vs. 67.8 %, *χ*^2^ = 3.46, *p* = 0.0630).

### Dosing, Hb, and safety outcomes

**Fig. 6:**
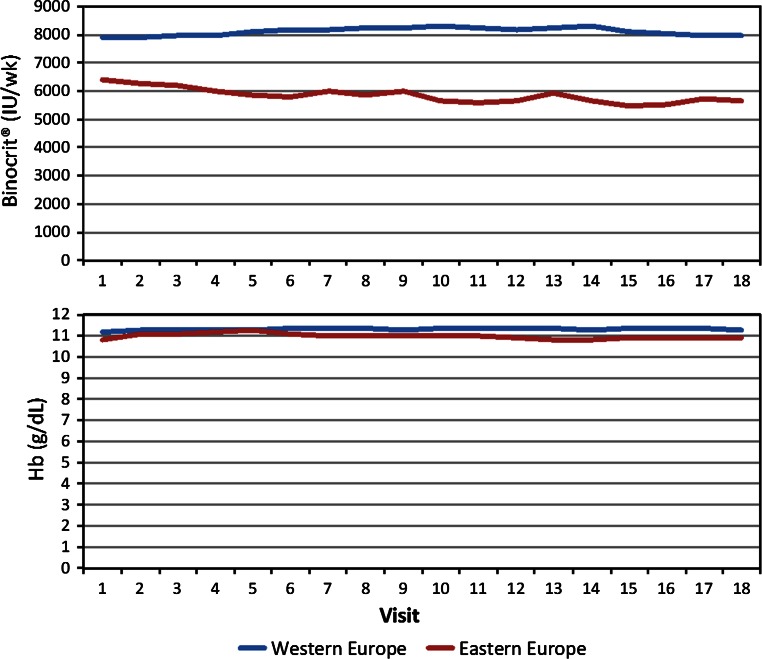
Mean Hb and biosimilar epoetin alfa dose by region

The majority of patients (82 %) were already receiving ESA therapy on study entry. Mean (SD) weekly dose of biosimilar epoetin alfa was 7532 (5342) IU, and mean (SD) Hb at baseline was 11.15 (1.17) g/dL. Dose of biosimilar epoetin alfa and Hb level over 18 months by region are illustrated in Fig. 6. Across all visits, patients in EE had significantly lower doses than patients in WE controlling for age and comorbidities (*β* = −0.2834, *p* < 0.0001; note that dose has been logarithmised, so the estimate represents percentage difference); and, while both EE and WE doses varied significantly over time (*β* = −0.0127, *p* < 0.0001 and *β* = −0.0034, *p* = 0.0370, respectively), the difference in trajectory was significantly different (*β* = −0.0093, *p* = 0.0042) with EE having a clear decline (from 6300 to 5500 IU/wk), while WE remained between 7900 and 8300 IU/wk but with month-to-month variability. Hb was also significantly different between regions controlling for age and number of comorbidities (*β* = −0.2114 g/dL, *p* < 0.0001) and with significantly different trajectories (*β* = −0.0165, *p* = 0.0002): EE had small but significant Hb decline (*β* = −0.0166, *p* < 0.0001), while WE had no significant change (*β* = −0.0001, *p* = 0.9618). At enrolment, 25 % of patients had experienced a prior thromboembolic event (TEE). During follow-up, 9.4 % of patients had a TEE, of which the majority were shunt thrombosis.

## Discussion

Our findings show that physicians’ attitudes towards anaemia management in haemodialysis patients have been influenced, at least in theory, by the findings of the TREAT study, conducted in patients with CKD not on haemodialysis. However, our data also demonstrate a discrepancy between these theoretical TREAT-influenced attitudes and actual practice patterns in terms of risk-based individualisation of target Hb levels and ESA dosing; while respondents claimed to use individualised target Hb levels for various risk groups, this is not reflected in patients’ actual Hb levels. The discrepancy at T2 (between theoretical upper Hb targets for specific risk groups and actual upper Hb targets at baseline) is particularly interesting given the apparent widespread adoption of KDIGO guidelines at this time point, which include probably the clearest recommendations yet on differential anaemia management. Thus while TREAT may have suggested the need for individualised Hb targets, which are also advocated in clinical practice guidelines, the clinical reality may be one of the ‘universally conservative’ Hb targets without risk stratification.

There are a number of possible explanations for the discrepancies between theoretical anaemia management and actual practice patterns. One is social desirability bias [9] which in the context of the current study might result in physicians giving the answers that they think are expected. Another possibility is that physicians are aware of the guideline recommendations but either have not translated them into their daily clinical practice or are outright sceptical of them. Further, as the European data from TREAT are different from the US data [4–10], European nephrologists may be doubtful of the TREAT findings and their generalisability to Europe. Time constraints may be another reason for the discrepancy between theory and practice; individualising treatment is time intensive, and it is easier/quicker to treat all patients according to established centre protocols. Reimbursement issues and increased use of bundling of care for haemodialysis patients may also contribute to lack of individualised anaemia management in actual practice. Another possible contributory factor is the influence of physicians’ training; for example, there are some data to suggest that US-trained doctors are more likely to be prepared to use higher ESA doses than their European counterparts [11].

The TREAT study raised particular concerns about use of ESAs in CKD patients with a history of cancer or stroke [4, 6]. The discrepancy in the present study between theoretical attitudes and actual practice patterns among these patient groups is noteworthy, given that more than 75 % of participating physicians indicated that results from the TREAT study (in patients not on dialysis) have influenced their use of ESAs in patients on haemodialysis, yet this is not evident in the actual Hb data.

Our data suggest a shift (at least in theory) to more aggressive use of iron supplementation in the management of anaemia in haemodialysis patients, as indicated by the increase at T2 in the proportion of physicians with a higher upper target for serum ferritin. This is consistent with reports from the Dialysis Outcomes and Practice Patterns Study, in which increasing use of intravenous iron and a rise in serum ferritin levels have been recorded [12, 13]. The increased use of iron to manage anaemia is likely to be reflective of more widespread use of bundling of care for haemodialysis patients, as well as a desire among physicians to limit the use of ESAs.

Significant regional variation in biosimilar epoetin alfa dosing was found; however, this did not translate into clinically significant differences in achieved Hb. While EE had approximately 28 % lower doses over the 18-month observation period, Hb was only 0.2 g/dL lower. Interestingly, patients in EE had significantly better iron stores than patients in WE but with no difference in supplementation rates. Nutritional, environmental, and/or genetic factors may contribute to this finding.

Data so far from the MONITOR-CKD5 study show that biosimilar epoetin alfa maintains stable longitudinal Hb outcomes, reflecting the same patterns as known with the originator epoetin alfa product. The data from MONITOR-CKD5 on Hb and dosing outcomes are also consistent with those from phase III and phase IV studies with this biosimilar epoetin alfa [14, 15].

## Conclusions

Physician investigators in the MONITOR-CKD5 study assert that their attitudes related to the use of ESAs in patients on haemodialysis have been influenced (at least in theory) by the results of the TREAT study, which involved patients not on dialysis. Physicians claim to use risk-based target Hb levels to guide renal anaemia care. However, there is discrepancy between these declared risk-based target Hb levels and actual target Hb levels for patients with variable risk factors. Further research is required to determine why the gap exists between hypothetical and actual target Hb levels.

## References

[1] Astor BC, Muntner P, Levin A (2002). Association of kidney function with anemia: the Third National Health and Nutrition Examination Survey (1988–1994). Arch Intern Med.

[2] KDIGO (2012). KDIGO clinical practice guideline for anemia in chronic kidney disease. Kidney Int Suppl.

[3] Locatelli F, Covic A, Eckardt KU (2009). Anaemia management in patients with chronic kidney disease: a position statement by the Anaemia Working Group of European Renal Best Practice (ERBP). Nephrol Dial Transplant.

[4] Pfeffer M, Burdmann EA, Chen CY (2009). A trial of darbepoetin alfa in type 2 diabetes and chronic kidney disease. N Engl J Med.

[5] Del Vecchio L, Locatelli F (2012). Safety issues related to erythropoiesis-stimulating agents used to treat anemia in patients with chronic kidney disease. Expert Opin Drug Saf.

[6] Skali H, Parving HH, Parfrey PS (2011). Stroke in patients with type 2 diabetes mellitus, chronic kidney disease, and anemia treated with darbepoetin alfa: the Trial to Reduce Cardiovascular Events with Aranesp Therapy (TREAT) experience. Circulation.

[7] Abraham I, MacDonald K (2012). Safety of erythropoiesis-stimulating agents in patients with end-stage kidney disease: data are safer than extrapolations. Expert Opin Drug Saf.

[8] Gesualdo L, London G, Turner M (2013). A pharmacoepidemiological study of the multi-level determinants, predictors, and clinical outcomes of biosimilar epoetin alfa for renal anaemia in haemodialysis patients: background and methodology of the MONITOR-CKD5 study. Intern Emerg Med.

[9] Phillips DI, Clancy KJ (1972). Some effects of ‘social desirability’ in survey studies. Am J Sociol.

[10] Locatelli F, Bárány P, Covic A (2013). Kidney Disease: Improving Global Outcomes guidelines on anaemia management in chronic kidney disease: a European Renal Best Practice position statement. Nephrol Dial Transplant.

[11] von Gersdorff G, Vega O, Schaller M et al (2011) Influence of age on anemia management: differences and commonalities between cohorts from a US and German registry. Abstract/poster from the XLVIII ERA-EDTA Congress, Prague, Czech Republic, June 23–26 2011. http://www.abstracts2view.com/era_archive/view.php?nu=ERA11L_1674. Accessed 21 Feb 2014

[12] Fuller D, Pisoni RL, Bieber BA (2013). The DOPPS practice monitor for US dialysis care: trends through December 2011. Am J Kidney Dis.

[13] Bailie GR, Larkina M, Goodkin DA (2013). Variation in intravenous iron use internationally and over time: the Dialysis Outcomes and Practice Patterns Study (DOPPS). Nephrol Dial Transplant.

[14] Haag-Weber M, Vetter A, Thyroff-Friesinger U (2009). Therapeutic equivalence, long-term efficacy and safety of HX575 in the treatment of anemia in chronic renal failure patients receiving hemodialysis. Clin Nephrol.

[15] Hörl WH, Locatelli F, Haag-Weber M (2012). Prospective multicenter study of HX575 (biosimilar epoetin-α) in patients with chronic kidney disease applying a target hemoglobin of 10–12 g/dl. Clin Nephrol.

